# Long-term follow-up of a female with congenital adrenal hyperplasia due to P450-oxidoreductase deficiency

**DOI:** 10.1590/2359-3997000000213

**Published:** 2016-09-26

**Authors:** Beatriz D. S. F. Bonamichi, Stella L. M. Santiago, Débora R. Bertola, Chong A. Kim, Nivaldo Alonso, Berenice B. Mendonca, Tania A. S. S. Bachega, Larissa G. Gomes

**Affiliations:** 1 Unidade de Endocrinologia Departamento de Medicina Irmandade da Santa Casa de Misericórdia de São Paulo São Paulo SP Brasil Unidade de Endocrinologia, Departamento de Medicina, Irmandade da Santa Casa de Misericórdia de São Paulo (ISCMSP), São Paulo, SP, Brasil; 2 Laboratório de Hormônios e Genética Molecular – LIM/42 Hospital das Clínicas Faculdade de Medicina da Universidade de São Paulo São Paulo SP Brasil Laboratório de Hormônios e Genética Molecular – LIM/42, Unidade de Adrenal, Disciplina de Endocrinologia e Metabologia, Hospital das Clínicas, Faculdade de Medicina da Universidade de São Paulo (HC-FMUSP), São Paulo, SP, Brasil; 3 Instituto da Criança Hospital das Clínicas Faculdade de Medicina da Universidade de São Paulo São Paulo SP Brasil Unidade de Genética, Instituto da Criança, Hospital das Clínicas, Faculdade de Medicina da Universidade de São Paulo (ICr-HC-FMUSP), São Paulo, SP, Brasil; 4 Centro de Pesquisa sobre o Genoma Humano e Células-Tronco Instituto de Biociências Universidade de São Paulo São Paulo SP Brasil Centro de Pesquisa sobre o Genoma Humano e Células-Tronco, Instituto de Biociências, Universidade de São Paulo (USP), São Paulo, SP, Brasil; 5 Departamento de Cirurgia Hospital das Clínicas Faculdade de Medicina da Universidade de São Paulo São Paulo SP Brasil Departamento de Cirurgia, Divisão de Cirurgia Plástica, Hospital das Clínicas, Faculdade de Medicina da Universidade de São Paulo (HC-FMUSP), São Paulo, SP, Brasil

## Abstract

P450 oxidoreductase deficiency (PORD) is a variant of congenital adrenal hyperplasia that is caused by POR gene mutations. The POR gene encodes a flavor protein that transfers electrons from nicotinamide adenine dinucleotide phosphate (NADPH) to all microsomal cytochrome P450 type II (including 21-hydroxylase, 17α-hydroxylase 17,20 lyase and aromatase), which is fundamental for their enzymatic activity. POR mutations cause variable impairments in steroidogenic enzyme activities that result in wide phenotypic variability ranging from 46,XX or 46,XY disorders of sexual differentiation, glucocorticoid deficiency, with or without skeletal malformations similar to Antley-Bixler syndrome to asymptomatic newborns diagnosed during neonatal screening test. Little is known about the PORD long-term evolution. We described a 46,XX patient with mild atypical genitalia associated with severe bone malformation, who was diagnosed after 13 years due to sexual infantilism. She developed large ovarian cysts and late onset adrenal insufficiency during follow-up, both of each regressed after hormone replacement therapies. We also described a late surgical approach for the correction of facial hypoplasia in a POR patient.

## INTRODUCTION

Congenital adrenal hyperplasia (CAH) comprises a group of autosomal recessive diseases that affect the synthesis of cortisol. Most CAH result from mutations in a single gene, which encodes a single steroidogenic enzyme. A new CAH variant due to mutations in the gene encoding the protein P450 oxidoreductase (POR), which is an electron donor to all the microsomal P450 enzymes type II, was first described in 2004 (1,2).

P450 oxidoreductase deficiency (PORD) causes partial and combined impairment of the key enzymes involved in steroidogenesis: P450c17(17α-hydroxylase/17,20 lyase), P450c21 (21-hydroxylase) and P450aro (aromatase). However, each enzyme may be differently compromised in a same patient (3,4). This variability in enzymatic impairments produces a wide phenotypic spectrum of clinical manifestations ranging from atypical genitalia in both sexes, adrenocortical insufficiency associated or not to bone malformations, to laboratory abnormalities in the neonatal screening test of asymptomatic newborns (1,4-8). A late onset form has also been reported in 46,XX patients with primary amenorrhea and polycystic ovarian morphology (1).

The ambiguous genitalia in both sexes is quite peculiar in PORD. Boys usually have incomplete external genitalia virilization due to impairment of 17,20 lyase activity, which decreases the testicular synthesis of androgens. In contrast, girls often have prenatal external genitalia virilization without postnatal hyperandrogenism. There are two hypotheses to justify this virilization in females. First, decreased placental P450c19 activity, which jeopardizes the conversion of fetal androgen precursors to estrogens. However, the 17,20-lyase activity is often impaired, which compromises the synthesis of fetal androgenic precursors by the classical pathway. Consequently, the most accepted hypothesis is the androgen production by an alternative pathway during fetal life, called the “backdoor pathway”, in which enzymes, such as SRD5A1 and AKR1C, convert 17OH-progesterone to dihydrotestosterone (DHT) (2,6,9).

It is expected that patients of both sexes will develop a collapse in the production of sex steroids during follow up because of the commitment impairments of P450c17 activity and P450c19 in women. However, there is only one report in the literature that describes the long-term evolution of seven patients who developed hypergonadotropic hypogonadism. In females, there were incomplete pubertal development and large ovarian cysts prone to spontaneous rupture (10).

Patients with PORD may also exhibit bone defects that are characteristics of Antley-Bixler syndrome because of the impairment of enzymes involved in the sterol synthesis pathway, such as lanosterol 14a-demethylase (*CYP51A1*), and enzymes involved in retinoic acid metabolism (*CYP26*) (11). Bone defects such as craniosynostosis, midface hypoplasia, radiohumeral and/or radio-ulnar synostosis, camptodactyly, and femoral steels were described (12). These skeletal malformations are observed in variable combinations and intensities, and sometimes they were only identified during development

Due to rarity of the disease and the data scarcity, we report one case of a 46,XX patient with mild atypical genitalia associated with severe bone malformations, highlighting the different approaches to medical treatment during puberty and the surgical management of midface hypoplasia.

### CASE REPORT

The patient was the second child born to consanguineous parents, natives of Juazeiro, Bahia, in Northeastern Brazil. The mother reported the development of acne and increased voice timbre during her first pregnancy, which progressed to fetal death in the eighth month of an unknown etiological factor. Morphological ultrasonography was not performed during first pregnancy. The mother developed similar hyperandrogenic signs in her second pregnancy.

The patient was born to term (birthweight of 2700g and height of 47cm), and atypical genitalia was not observed at birth, only the presence of severe bone abnormalities in the face and upper limbs. Postnatal adaptation passed well, and there were no reports of neonatal dehydration.

The patient developed pubarche at 11 years old and thelarche at approximately 11.5 years old. She was referred to our department when she was approximately 13 years old for investigation of primary amenorrhea and bone malformations.

Physical examination revealed a height of 154 cm (SD 0), target height was 160.8 cm, sitting height of 79 cm (SD 0), weight 29.6 kg (SD -1,63), blood pressure of 112 x 78 mmHg, breasts Tanner II, pubic hair Tanner III, clitoromegaly 2 cm, and a posterior fusion of major labia (Prader II). Microbrachycephaly, midface hypoplasia, proptosis, depressed nasal bridge, pear-shaped nose, dysplastic and protruding ears, severe elbow restriction and limited upper limb motion, arachnodactylyin hands, shortening of the 4^th^ and 5^th^ toes were also observed. The patient had normal cognition and school performance.

Complementary exams revealed: skeletal survey with radio-humeral synostosis, carpal bone fusion, bone age (BA) of 10 years old, shortening of the 4^th^ and 5^th^ metatarsus, scoliosis, exostosis in clavicles; 3D cranial CT scan showed pansynostosis; normal echocardiogram and abdominal ultrasound with mild right pyelectasia.

The initial hormonal investigation found moderately increased serum 17OHP levels and markedly increased progesterone levels at baseline and after an ACTH-stimulation test (250 mcg i.v.). Basal serum cortisol levels were normal, but an impaired response was observed in the ACTH-stimulation test. Serum androgen levels (androstenedione, testosterone and DHEA) were low at baseline and in the ACTH test. The precursors 17OH-progesterone and progesterone were moderately and very elevated (Table 1). Basal FSH was 12,7 UI/L (normal range Tanner II until 3.9 UI/L, immunofluorimetric assay), LH was 19,2 UI/L (normal range for Tanner II until 1,2 UI/L, immunofluorimetric assay) and estradiol was 17 pg/mL (pubertal value > 22 pg/mL, fluorimmunoassay), consistent with hypergonadotropic hypogonadism.

**Table 1:** Basal and ACTH-stimulated adrenal steroid levels

Time (min)	RV	-60	0	60
Progesterone (ng/mL)	0,4-1,1	25.2	16.5	71.1
17OHP (ng/mL)	0-1,1	20.7	9.7	32.4
Androstenedione (ng/mL)	0-2,2	1.0	0.7	0.8
DHEA (ng/mL)		< 1	1	1.1
Cortisol (µg/dL)	6,7-22,6	12.6	8.4	11.6
Testosterone (ng/dL)	< 12-48	< 12		

Progesterone and testosterone were measured by fluoroimmunoassay, 17OH-progesterone by radioimmunoassay (17OHP) and androstenedione, cortisol by chemiluminescence.

RV: reference values for the female follicular phase.

DNA samples were extracted from peripheral blood and submitted to PCR amplification of *POR *exonic regions and direct sequencing. The p.A287P mutation was identified in a homozygous state in the patient and heterozygosis in her parents. Multiplex Dependent Probe Amplification-ligands (MLPA) did not identify changes in the *POR *gene copy number.

Pelvic ultrasound revealed an infantile uterus and bilateral polycystic ovaries, with the largest cyst measuring 2.8 cm in the right ovary. Full right ovarian volume was 17.5 mL, and left ovarian volume was 5.3 mL. Conjugated estrogens (0.3 mg/day) were introduced due to the absence of menarche at age 14, and menarche occurred when she was 14.5 years old. The next ultrasound image revealed a complete regression of ovarian cysts. The conjugated equine estrogens were replaced with combined treatment with estradiol valerate (2 mg) and levonorgestrel (medications available with our Public Health System). Breast development improved during the last year of follow-up, and she is presenting regular menstrual cycles under estrogenic replacement therapy. Low bone mineral density (BMD) was also observed: total femur 0.634 g/cm^2^ (Z -2.8), femoral neck of 0.617 g/cm^2^ (Z -2.1) and L1-L4 of 0.676 g/cm^2^ (Z -2.4).

At 15 years old, she underwent plastic surgery to correct the severe maxillary hypoplasia using external facial distraction osteogenesis. First, she was submitted to facial advancement with a Le Fort III osteotomy and mobilization of the midface forward until correction of class III malocclusion; this was a gradual advancement with the RED (rigid external device from KLS Martin). In a second procedure tissue expander was used, followed by acrylic molded implant to improve the projection of frontal area. The last procedure for refinements was rhinoplasty to adjust the facial harmony (Figure 2). The patient received the stress glucocorticoid dose at each procedure.

**Figure 1:**
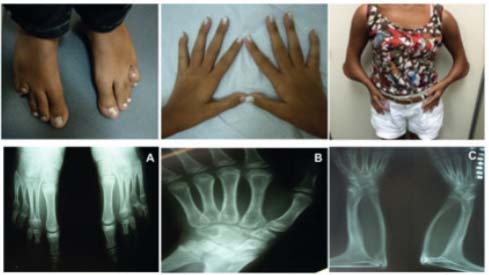
Skeletal abnormalities in 46,XX patient with POR deficiency. **(A) **Shortening of the 4^th^ and 5^th^ metatarsus; (**B**) arachnodactyly and carpal bone fusion; (**C**) Severe elbow restriction with radio-humeral synostosis.

**Figure 2:**
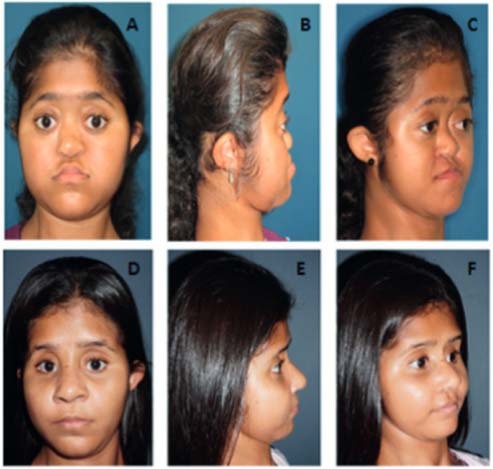
Facial characteristics before (A,B,C) and after (D,E,F) surgery: Le Fort III advancement.

The patient received glucocorticoid stress dose in the first facial surgery for 5 days. After the glucocorticoid withdrawal, she developed severe chronic fatigue and drowsiness that improved with the reintroduction of glucocorticoid therapy (dexamethasone solution 0.1 mg/day).

### DISCUSSION

There has been a growing increase in descriptions of patients with P450-oxidoreductase deficiency since its first reports in 2004, and this disorder is characterized by wide phenotypic variability. However, few reports emphasize the clinical manifestations in correlation with genotypes and outcomes during puberty and adulthood. We describe the manifestations of our case and previous reports.

Our patient carried the p.A287P mutation in homozygosis, which is the most frequent mutation in Caucasians, and it is observed in approximately 43% of nonrelated alleles (8). Our case, and other 46,XX patients who are homozygous for this mutation, presented with external genitalia virilization, in contrast to 46,XY patients who exhibit normal external genitalia. On the other hand, the most common mutation in the Japanese population, the p.R457H mutation, is associated with a higher prevalence of atypical genitalia in both 46,XX than 46,XY patients (100% versus 44%, respectively) (13). The degree of external genitalia virilization is moderate in most 46,XX PORD patients, which is differently than the 21-hydroxylase deficiency. However, 46,XX cases presenting with severe virilization (Prader scores IV and V) including wrong sex assignment have also been described (7,14).

Our patient presented late onset glucocorticoid deficiency. Basal serum cortisol levels were normal, but impaired cortisol secretion was observed after ACTH-stimulation. The adrenal insufficiency documented by cosyntropin was found in almost all patients in large cohorts, of those 50% needed permanent hydrocortisone (8,13). This phenotype is due to impairment of P450c17 activity with a concomitant deficiency of P450c21. Consequently, these patients may present with moderately increased basal 17-hydroxyprogesterone (17OHP) levels, but these levels are not as high as the classical form of 21-hydroxylase deficiency. This increase may be confused with the diagnosis of 21-hydroxylase deficiency in some situations. P450c17 deficiency is consistent with increased 21-deoxycortisol secretion, which protects against the development of adrenocortical insufficiency in the first years of life in most cases. Notably, high blood pressure due to increased DOCA and impairment of 17α-hydroxylase was reported (1).

There are few data on puberty development (10,13,15). Our case presented partial pubertal development at age of 14, which was similar to the previous series of seven PORD patients, in which five female patients presented pubertal delay and large ovarian cysts were observed in all of them (10). The insufficient pubertal development and amenorrhea is mainly explained by P450c17 impairment. In our cohort of 46,XX patients with P450c17 deficiency, primary amenorrhea (70%), lack of breast development (50%) and sparse or absence of pubic hair (100%) were the most prevalent findings. Spontaneous breast development until Tanner IV was a very rare finding (16). One hypothesis for the prevalence of ovarian cysts includes the increased LH stimulation due to primary hypogonadism; which is not supported by the description of a patient with amenorrhea, ovarian cysts and inactivating mutations of the LH receptor gene (17). Another hypothesis may be the negative impact of PORD on the synthesis and metabolism of steroids involved in oocyte maturation. The *CYP51A1* requires the transfer of electrons by the POR for its catalytic activity, which promotes the conversion of lanosterol to meiosis-activating sterols. Follicular fluid meiosis-activating sterols are essential to promote resumption of oocyte meiosis during puberty and support oocyte maturation (18,19). The treatment with estrogen/progestin replacement was efficient to promote cysts regression and to avoid new cysts formation, probably through decreased LH stimulus, confirming previous study (10). In addition, the estrogen/progestin replacement was crucial to keep regular menstrual cycle and improve bone mass.

The bone malformation phenotype presents the highest variability in PORD patients. Our case presented pancraniosynostosis, maxillary hypoplasia, depressed nasal bridge, bilateral elbow synostosis, femoral bowing and camptodactyly in hands and feet consistent with severe phenotype based on PORD malformation score (8). However, the six patients report by Krone and cols. homozygous for p.A287P had mild to moderate malformations (8). Patients with more severe bone malformation phenotype harbored at least one null mutation in one allele, but in our patient this was not confirmed; and the genetic factors involved in these variations in bone phenotype are not known. The ideal age for correction of midface hypoplasia and craniosynostosis to allow adequate brain development is between 6 months to 1 year old (20); even though in this particular patient surgical procedures were late, they correct morphologic abnormalities of face, orbit and jaw. Notably, the patient had great facial cosmetic result with improvement of sociability and relationships with peers.

The PORD phenotype is not restricted to the patient. Hyperandrogenic manifestations in mothers are described during pregnancies of PORD fetuses and range from acne to hirsutism until virilization signs appear, such as a deepening of the voice, which was observed in the mother of our patient. However, there is no data in the literature on the frequency of maternal manifestations and their correlation with specific POR mutations. A single multicenter study of 20 pregnant women found that the presence of maternal virilization and severe bone defects detected by fetal ultrasound may be the fundamental characteristics of fetuses with PORD (7). We speculated that the mother’s hyperandrogenic manifestations at the first pregnancy may have been reflective of PORD, since the risk for an affected child for this couple is 25%.

In conclusion, PORD is a rare disease with significant phenotypic variability that affects multiple systems. We described the second PORD case in our population (14), and provided a warning of this diagnostic possibility in patients with genital ambiguity and low serum androgen levels. We stressed the importance of continuous monitoring for glucocorticoid deficiency, the need for hormone replacement therapy with sex steroids at puberty and the benefit of the late surgical approach in facial hypoplasia.
